# Glycemic Control and the Weight Benefit of a Daily 7 mg Dose of Oral Semaglutide Versus an Alternate-Day 14 mg Dose of Oral Semaglutide From an Ambulatory Glucose Monitoring Data: A Retrospective Cohort Study From Eastern India

**DOI:** 10.7759/cureus.40179

**Published:** 2023-06-09

**Authors:** Soumyabrata RoyChaudhuri, Anirban Majumder, Poulomi Mukherjee, Debmalya Sanyal, Soma Chakraborty, Susama Chuyan

**Affiliations:** 1 Diabetes and Endocrinology, Kali Prasad Chowdhury Medical College and Hospital, Kolkata, IND; 2 Community Medicine, Medical College and Hospital, Kolkata, IND; 3 Diabetes and Endocrinology, Diabetes-Obesity-Thyroid-Hormone Clinic, Kolkata, IND; 4 Diabetes and Endocrinology, Adopt Endocrine, Kolkata, IND

**Keywords:** types 2 diabetes, glycaemic control, body mass index (bmi), ambulatory glucose profile (agp), oral semaglutide

## Abstract

Introduction: Oral semaglutide is a glucagon-like peptide-1 receptor agonist (GLP-1RA) class of antidiabetic medication. High costs and GI side effects are the major limitations of its widespread use. Some patients who were on a 14 mg dose of oral semaglutide self-prescribed an alternate-day schedule to mitigate GI side effects and to reduce the cost.

Methods: This retrospective observational cohort study evaluates the ambulatory glucose profile (AGP) data, extrapolated glycosylated hemoglobin (HbA1C), and BMI of 11 types of 2 diabetes mellitus (T2DM) while they were on an alternate-day 14 mg dose of oral semaglutide compared to their record while on a daily 7 mg dose. The AGP metrics (time-in-range (TIR), time-below-range (TBR), and time-above-range (TAR)) along with extrapolated HbA1C and BMI were analyzed. Statistical analysis was done using SPSS Statistics version 21.0.

Results: No statistically significant difference in the AGP metrics between the AGP profile of a daily 7 mg dose and the AGP profile of an alternate-day 14 mg dose of oral semaglutide was observed. Interestingly, a statistically significant progressive decline in BMI value was observed even on the alternate-day 14 mg dose when compared to the daily 7 mg dose.

Conclusion: In this small cohort of patients, the metrics of short-term glycemic control and the extrapolated HbA1C values were similar for the daily 7 mg dose versus the alternate-day 14 mg dose of oral semaglutide. BMI showed progressive reduction which was statistically significant even with the alternate-day 14 mg dose of oral semaglutide.

## Introduction

Glucagon-like peptide-1 receptor agonists (GLP-1RAs) were approved for the treatment of type 2 diabetes mellitus (T2DM) in 2005 [1]. Its robust glycosylated hemoglobin (HbA1c) lowering efficacy along with its low potential for causing hypoglycemia and weight reduction benefits gradually pulled the drug up the ladder in the management algorithm of T2DM. The American Diabetes Association (ADA) 2018 formally placed GLP-1RA as the first injectable drug in the treatment of T2DM ahead of insulin [2]. Liraglutide, dulaglutide, albiglutide, and semaglutide, the injectable formulations of GLP-1RA, were found safe or beneficial according to cardiovascular outcome trial results (LEADER, HARMONY, REWIND, and SUSTAIN-6) [3-6]. However, they failed to receive global acceptance as a result of clinical inertia due to three primary reasons: (a) cost of therapy, (b) injectable mode of administration, and (c) GI side effects [7].

Approval of the oral version of semaglutide in 2019 by the United States Food and Drug Administration was a game changer. In this oral version, semaglutide (peptide) was co-formulated with salcaprozate sodium or SNAC [sodium N-(8-{2-hydroxybenzoyl} amino) caprylate]. SNAC, an absorption enhancer, caused a transient rise in the local pH of the stomach, which protected the oral peptide from proteolytic degradation and also facilitated the absorption of the drug via the trans-cellular route across the gastric epithelium in a concentration-dependent manner [8-10]. The oral semaglutide reaches a steady state concentration 4-5 weeks after the start of therapy. Once absorbed, its pharmacokinetics and pharmacodynamics are exactly similar to that of once-weekly [QW] injectable s/c semaglutide with a half-life of one week [11].

Oral semaglutide was made available in India for use from January 2022. Some of the patients by their ingenuity devised a self-made protocol of an alternate-day 14 mg dose of oral semaglutide to alleviate the GI symptoms and to reduce the cost (presumed but not directly disclosed by any patient). The self-monitoring of the blood glucose log of these patients looked identical on days of oral semaglutide usage (days-on-drug) versus the days of voluntary drug abstinence (days-off-drug) [12]. This finding led us to compare their ambulatory glucose profile (AGP) data during the daily 7 mg dose of oral semaglutide and AGP data during the alternate-day 14 mg dose of the same patient.

## Materials and methods

T2DM patients were prescribed oral semaglutide from the Endocrinology Outpatient Department of Kali Prasad Chowdhury Medical College and Hospital as per ADA management protocol after screening the retina for diabetic retinopathy. AGP using the Freestyle Libre Pro sensor (Abbott, Illinois, United States) was advised to all patients as a standard operating procedure who were prescribed oral semaglutide. AGP was conventionally done when they were on a stable dose of 7 mg of oral semaglutide for at least four weeks and then again after up-titration when they were on a stable dose of 14 mg for at least four weeks. This retrospective observational real-world cohort study examined the 14-day AGP data of the patients on a daily 7 mg dose of oral semaglutide and the AGP data of the same patients if they had adopted an alternate-day 14 mg dose. The glycemic control of the two sets of AGP data was compared for the glycemic metrics: time-in-range (TIR), time-below-range (TBR), time-above-range (TAR), and extrapolated HbA1C.

The inclusion criteria included patients with T2DM on oral semaglutide, who received a daily 7mg dose of oral semaglutide for at least six weeks followed by an alternate-day 14 mg dose for at least another six weeks, who performed an AGP for 14 days after receiving a daily 7 mg dose for at least four weeks and again after receiving an alternate-day 14 mg dose for at least four weeks, with unchanged antidiabetic medications during the entire period of observation, and with serious acute illness during the entire period of observation.

The exclusion criteria included patients with T2DM on oral semaglutide on irregular follow-up, who were pregnant, and who were diagnosed with type 1 diabetes mellitus or latent autoimmune diabetes of the adult.

After browsing through the database, 11 patients were identified who fulfilled all the inclusion and exclusion criteria. Their AGP data including extrapolated HbA1C obtained during a daily 7 mg dose and an alternate-day 14 mg dose of oral semaglutide were taken up for analysis. Any change in the recorded BMI was also analyzed. The study was retrospective and observational in nature; thus, ethics committee approval was not sought. Anonymity and confidentiality were strictly observed and bioethics-related tenets pertaining to the Helsinki Declaration were strictly adhered to. Statistical Analysis System (SAS) version 9.2 for Windows (SAS Institute Inc, Cary, NC, USA) and SPSS Statistics version 21.0 for Windows (IBM Corp. Released 2012. IBM SPSS Statistics for Windows, Version 21.0. Armonk, NY: IBM Corp.) were used for statistical analysis.

## Results

Eleven patients were undertaken for analysis, seven were male and four were female, average age was 58 ± 11 years, BMI was 32± 4.7 kg/m2, and HbA1c was 7.7±1.8% (Table 1).

**Table 1:** Baseline characteristics of the overall cohort (at the start of AGP on a daily 7 mg dose) BMI: body mass index, HbA1c: hemoglobin A1C

Parameters	Mean	Standard deviation
Age (years)	58	11
Sex (male:female)	1.6	0.5
BMI (kg/m^2^)	32	4.7
HbA1c (%)	7.7	1.8

As the sample size was small (n=11), the data of TIR, TBR TAR, and extrapolated HbA1c were tested for the normality of distribution by the Shapiro-Wilk test which showed that the variables were not normally distributed. Hence, a non-parametric Mann-Whitney U test was performed to compare the two groups' differences. The Mann-Whitney U test was applied to the parameters (as the set of data obtained had a non-normal distribution): (1) HbA1C (extrapolated values during the 14 days of AGP monitoring), (2) TIR, (3) TBR, and (4) TAR. No statistically significant differences were found between extrapolated HbA1c, TIR, TBR, and TAR in the Mann-Whitney U test as the p-values were more than alfa viz 0.05 (Table 2).

**Table 2:** Change in parameters observed in a daily 7 mg dose versus an alternate-day 14 mg dose of oral semaglutide *p-value <0.05 considered significant, p calculated by Mann Whitney U test HbA1C: hemoglobin A1C, TIR: time-in-range, TBR: time-below-range, TAR: time-above-range

Parameters	Mann-Whitney U test	Z	Sig. (two-tailed)
HbA1C (%)	43	-1.157	0.247
TIR (mg/dl)	43.5	-1.118	0.264
TBR (mg/dl)	37.5	-1.528	0.126
TAR (mg/dl)	53	-0.528	0.598

Change in BMI in the interim period, which is a continuous variable, proved to be statistically significant between the application of the first AGP sensor and the completion of the 14 days of the second AGP sensor, with a p-value of <0.003 (Table 3).

**Table 3:** Change in BMI Continuous variables are expressed in Mean (SD), * for p-value which is significant BMI: body mass index

Parameter	Mean	Standard deviation	p-value
BMI (kg/m^2^)	30	3.9	0.0003*

## Discussion

Alternate-day dosing of drugs having a long half-life may have a profound impact in terms of cost if they demonstrate similar efficacy on alternate-day dosing compared to daily dosing. Long-acting statins (atorvastatin and rosuvastatin) on alternate-day dosing were found to be safe and efficacious [13,14]. Similarly, the alternate-day dose of linagliptin, a long-acting dipeptidyl peptidase 4 (DPP4) inhibitor class of anti-diabetic medication, was found to have acceptable fasting plasma glucose (FPG), postprandial plasma glucose (PPPG), and HbA1c levels compared to the daily dose [15]. This is of paramount importance in the present context: as the primary reason for non-adherence to the prescribed drug regimen, especially in the case of chronic disorders, the cost of medication is increasing [16-19]. In our previous study on the alternate-day 14 mg dose of oral semaglutide, the average values of TIR and TBR between the days-on-drug versus the days-off-drug had no statistically significant difference [12].

Intensive glucose lowering, targeting the HbA1c, had failed to demonstrate a reduction of macrovascular event rate or mortality in T2DM in four long-term randomized open-labeled trials, namely Action to Control Cardiovascular Risk in Diabetes, Action in Diabetes and Vascular Disease: Preterax and Diamicron Modified Release Controlled Evaluation, Veterans Affairs Diabetes Trial, and United Kingdom Prospective Diabetes Study [20-23). In this context, AGP emerged as a complementary reliable therapeutic target for patients with T2DM [24,25]. TIR is used as a short-term measure of glycemic control, and a target range of 70-180 mg/dl for non-pregnant T2DM was proposed. Efforts have been on to correlate the TIR values with that of HbA1c [26]; however, a uniform correlation coefficient is yet to be derived. Dixon et al. in a cohort of 1924 people with T2DM proposed a correlation coefficient of -0.78 [27]. Kesavadev et al. reported that the HbA1c of < 7.5% in Asians corresponds to a TIR value of > 70% [28].

In our cohort of 11 patients with T2DM, the AGP data was made available while on a daily 7 mg dose and while on an alternate-day 14 mg dose of oral semaglutide. Interestingly, the average TIR on the alternate-day 14 mg dose of semaglutide (85.81±12.75 %) was higher than the average TIR on a daily 7 mg dose (75.09±23.75%). However, this difference was not statistically significant. The average TBR and TAR values between a daily 7 mg dose and an alternate-day 14 mg dose of semaglutide dose showed no statistical significance. HbA1c was not repeated within this short interim period. Nevertheless, we compared the extrapolated HbA1c given by the Freestyle Libre Pro sensor system (by analyzing the 14-day data) between the same set of patients, once while on a daily 7 mg dose of oral semaglutide and also while on an alternate-day 14 mg dose, and found no statistically significant difference. BMI, recorded at the beginning of the first AGP monitoring (on a daily 7 mg dose) and at the end of the second AGP monitoring (on an alternate-day 14 mg dose), showed a statistically significant reduction from 32±4.7 kg/m2 to 30±3.9 kg/m2.

Limitations

The study has many limitations. First, the study has a small number of patients. Second, observation was done for a very brief period. Third, FPG, PPPG, and HbA1c, the conventional parameters of glycemic control, were not compared to further justify similar glycemic control with a daily 7 mg dose versus an alternate-day 14 mg dose of oral semaglutide. Fourth, other pleiotropic benefits of the glucagon-like peptide-1 (GLP-1) class of molecule, especially cardiovascular outcomes, were not investigated.

## Conclusions

The increasing cost of medication, especially for long-term therapy, is a major hurdle for patient adherence. Oral semaglutide is a novel GLP-1RA class of antidiabetic drug that contains peptides within a pill but comes at a premium cost. Our observation on the AGP profile of patients on the alternate-day 14 mg dose of oral semaglutide suggests that similar glycemic patterns were achieved compared to the daily 7 mg dose schedule. There was continued weight loss on the alternate-day dosing schedule also. However, a longer period of study with a larger study population along with other pleiotropic benefits as the secondary outcome is required before we can propose alternate-day dosing as an effective and also economical therapeutic option for patients with T2DM.
